# Genetic variability in Italian populations of *Drosophila suzukii*

**DOI:** 10.1186/s12863-017-0558-7

**Published:** 2017-11-03

**Authors:** Gabriella Tait, Silvia Vezzulli, Fabiana Sassù, Gloria Antonini, Antonio Biondi, Nuray Baser, Giorgia Sollai, Alessandro Cini, Lorenzo Tonina, Lino Ometto, Gianfranco Anfora

**Affiliations:** 1 Research and Innovation Centre, Fondazione Edmund Mach, San Michele all’Adige, Trento, Italy; 2 Department of Agricultural and Environmental Sciences, Udine Univeristy, Udine, Italy; 3 Division of Nuclear Techniques in Food and Agriculture, FAO/IAEA, Wien, Austria; 4 “Charles Darwin” Department of Biology and Biotechnologies, Sapienza University, Rome, Italy; 5 Department of Agriculture, Food and Environment, Catania University, Catania, Italy; 6 Mediterranean Agronomic Institut, Valenzano, Bari, Italy; 7 Department of Biomedical Science, Cagliari University, Cagliari, Italy; 8 Centre for Biodiversity and Environment Research, London College University, London, UK; 9 Department of Agronomy, Padova University, Padova, Italy; 10 Center of Agriculture Food Environment, Trento University, San Michele all’Adige, Trento, Italy; 11 Current address: Independent Researcher, Mezzocorona, Trento, Italy

**Keywords:** Spotted wing drosophila, SSR markers, Population structure, Gene flow, Bottleneck, Human trade

## Abstract

**Background:**

*Drosophila suzukii* is a highly destructive pest species, causing substantial economic losses in soft fruit production. To better understand migration patterns, gene flow and adaptation in invaded regions, we studied the genetic structure of *D. suzukii* collected across Italy, where it was first observed in 2008. In particular, we analysed 15 previously characterised Simple Sequence Repeat (SSR) markers to estimate genetic differentiation across the genome of 278 flies collected from nine populations.

**Results:**

The nine populations showed high allelic diversity, mainly due to very high heterozygosity. The high Polymorphism Information Content (PIC) index values (ranging from 0.68 to 0.84) indicated good discrimination power for the markers. Negative fixation index (*F*_IS_) values in seven of the populations indicated a low level of inbreeding, as suggested by the high number of alleles. STRUCTURE, Principal Coordinate and Neighbour Joining analysis also revealed that the Sicilian population was fairly divergent compared to other Italian populations. Moreover, migration was present across all populations, with the exception of the Sicilian one, confirming its isolation relative to the mainland.

**Conclusions:**

This is the first study characterising the genetic structure of the invasive species *D. suzukii* in Italy. Our analysis showed extensive genetic homogeneity among *D. suzukii* collected in Italy. The relatively isolated Sicilian population suggests a largely human-mediated migration pattern, while the warm climate in this region allows the production of soft fruit, and the associated *D. suzukii* reproductive season occurring much earlier than on the rest of the peninsula.

**Electronic supplementary material:**

The online version of this article (10.1186/s12863-017-0558-7) contains supplementary material, which is available to authorized users.

## Background

The spotted wing drosophila (SWD), *Drosophila suzukii* Matsumura (Diptera: Drosophilidae), is a pest species which has spread from its original range in Asia to a number of western countries in the past decade, including the Mediterranean basin [1], Europe, and USA [1–3]. The history of the geographical spread and infestation of *D. suzukii* is still under investigation: it is known that in 1939 this species was first recorded in Japan (Kanzawa 1939), while in the 1980s it was collected on the island of Hawaii [4]. Europe and the Americas were colonised much later, possibly during the last 9 years [2, 3, 5, 6]. First adults of *D. suzukii* were caught contemporaneously in the region of Catalonia, Spain [7] and in Tuscany, Central Italy, in 2008 [3]. In 2009 *D. suzukii* individuals were found on both wild hosts (*Vaccinium*, *Fragaria* and *Rubus* spp.) and several species of cultivated berries in Trento Province, North Eastern Italy, where also the first economically important damage by this species in Europe was reported [8]. During the following years, *D. suzukii* has been spreading rapidly across Europe, with documented infestations ranging from Mediterranean regions (i.e. Greece, Turkey) to northern latitudes (i.e. Sweden, Poland, UK) (EPPO Global Database, *Drosophila suzukii* – DROSSU, 2017). In Italy, after the first detection, infestations were reported from the regions of Bolzano, Piedmont, Liguria, Campania and Veneto in 2010, from Lombardy, Emilia Romagna, Marche, Aosta Valley, Marche, Calabria and Sicily in 2011 [3], Sardinia in 2012 [9], Apulia in 2013 [10], Umbria in 2014 [11], and Latium in 2015 (Antonini G., present paper). Invasion dynamics can be studied using molecular markers that can discriminate and characterise the genetic relationships between source and derived populations, migration flows and population expansion patterns [12–14]. In particular, Single Nucleotide Polymorphism (SNP) and Simple Sequence Repeat (SSR) markers have played an increasingly significant role in the study of genetic differentiation across species populations [15]. Thanks to their great discrimination power and high reproducibility and variability, SSRs represent one of the most robust and informative molecular markers available for genotyping individuals [16]. For instance, their use in *Drosophila* species was pivotal in studying intra-population genetic variation and evolution [14, 17–20].

In relation to *D. suzukii*, SSRs have been exploited to study genetic aspects of the colonisation process in the USA and Europe. Jeffrey and colleagues based their research on the use of six X-linked genes and suggested that the invasions of the USA and Europe are two independent events [21]. Bahder et al. in particular analysed samples of *D. suzukii* populations collected in California and Washington and determined that while *D. suzukii* in the former region had high levels of genetic variation, the latter was highly monomorphic [22]. Furthermore, Fraimout’s group investigated Hawaiian and Spanish populations by exploiting microsatellite markers, finding a significant level of genetic differentiation [23]. Although both studies exploited two different sets of microsatellites and tested different populations, the authors were led to similar conclusions: they demonstrated the presence of a specific differentiation process among ancestral and derived populations and suggested that for *D. suzukii* a genetic analysis approach is valuable not only to better understanding of the evolutionary history of the species, but also to manage its great potential for invasiveness. Different studies on the invasiveness of species, including *D. suzukii*, have demonstrated the relationship between their spread and human trade [24–26]. For this reason, it is very important to consider the correlation between gene flow analysis and the sale of soft fruit all over the country. Taking into account this aspect, we used a population genetic approach to characterise genetic diversity among *D. suzukii* individuals collected in different regions of Italy. In order to perform this work a set of 15 microsatellites validated by Fraimout and colleagues [23] were employed. The current research is the first study that provides new insights on the trend of genetic diversity in Italian populations of *D. suzukii*.

## Methods

### D. Suzukii collection, identification and DNA extraction

**Fig. 1:**
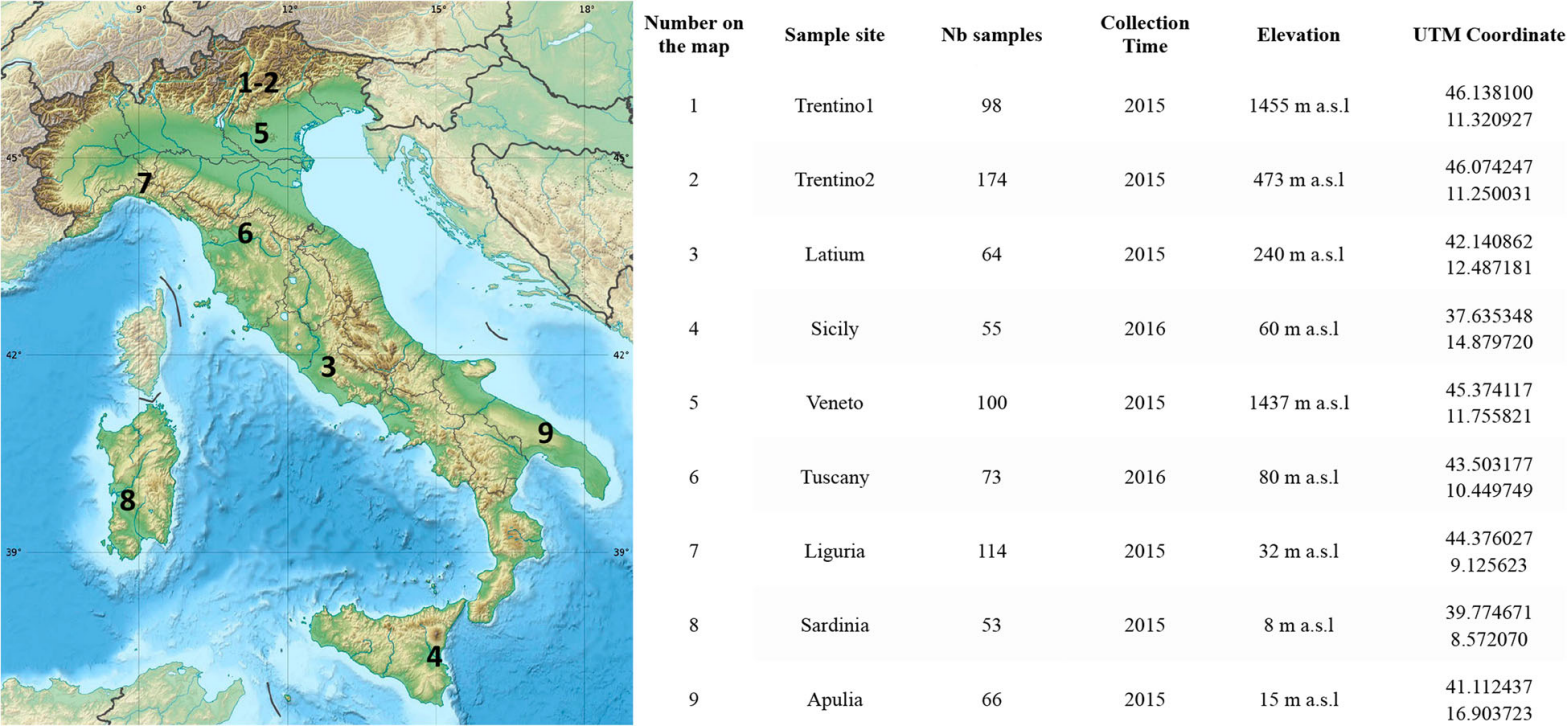
Field collected samples of the *D. suzukii* analysed in this study. This image has been adapted from the original (https://it.wikipedia.org/wiki/File:Italy_topographic_map-blank.svg) whose author is Eric Gaba, and it is licensed through Creative Commons Attribution 3.0

A total of 278 individuals of *D. suzukii* collected from nine populations in Italy were analysed (Fig. 1). Adult *D. suzukii* were collected between October 2015 and April 2016 using Droskidrink®-baited traps [27] left exposed for 3 days. In order to limit the likelihood of sampling individuals related to each other, three traps per location were used, at a distance of at least 500 m from each other. In the laboratory, *D. suzukii* individuals were identified using a 7×-45× stereomicroscope, according to Hauser’s (2011) morphological characteristics, such as the structure of the ovipositor for females and spots on the wings and tarsal combs for males. Samples were preserved in 96% ethanol and kept at 4 °C until DNA extraction. For each location, we selected 15 females and 15 males for DNA extraction, with genomic DNA being extracted from each individual separately using the Macherey Nagel Kit (NucleoSpin Tissue, Macherey Nagel, Düren, Germany).

### Microsatellite analysis

**Fig. 2:**
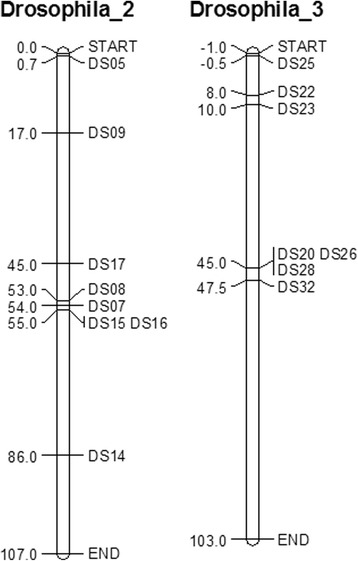
Location of the 15 microsatellite markers distributed across the chromosomes 2 and 3

The SSRs used for this work were selected from a set of microsatellites previously designed and validated [23]. Of the 28 published SSRs, 22 continuous di-nucleotide loci were tested on a pool of 20 *D. suzukii* individuals. Seven of these loci were discarded because of amplification problems, leaving 15 SSR markers distributed across chromosomes 2 and 3 (Fig. 2) [28].

Each pair of primers was used for PCR amplification in 25 μL final volume, containing 1X GoTaq G2 Master Mix, 0.5 μL of each primer, 10.5 μL of distilled deionized water and 1 μL of genomic DNA. The PCR program was set with an initial period of denaturation at 94 °C (30 s) followed by 32 cycles of additional denaturation at 94 °C (30 s), an annealing phase at 57 °C (1 min 30 s), an elongation phase at 72 °C (1 min), and ending with another extension phase at 72 °C (30 min). PCR products were checked using electrophoresis on 1.5% agarose gel, stained with ethidium bromide and visualised under UV light. Each amplicon was then diluted 1:10 in distilled water and 1 μL of this dilution was added to 12.5 μL of a mixture of deionised Formamide (Sigma-Aldrich) and GeneScan-500 ROX size standard (Life Tech, Waltham, MA USA). Prior to denaturation for 4 min at 94 °C, capillary electrophoresis was carried out in an ABI PRISM 310 Genetic Analyzer (Life Tech) and the fragments were sized with GeneMapper v.4.0 software in binning mode. If no sample amplification was obtained after two PCR attempts, the locus was classified as missing data.

### Statistical analysis

Microsatellite allele data were processed with Tandem program v.1.08 [29]. GenAIEx software v.6.41 [30] was run to study the genetic variability between populations using the following statistics: mean number of alleles (*N*_a_), effective number of alleles (*N*_e_), expected heterozygosity (*H*_E_), observed heterozygosity (*H*_O_), number of private alleles (*N*_p_), frequency of private alleles (*A*_p_) and inbreeding coefficient (*F*_IS_). Allelic richness was calculated using FSTAT v.2.9.3 software [31]. Deviation from Hardy-Weinberg equilibrium after the Bonferroni multiple correction test and allelic Polymorphic Information Content (PIC) were tested using CERVUS software v.3.0 [32]. *N*_e_ and *H*_E_ were chosen as the basic genetic variability and estimated for each population. *N*_e_ was analysed with ANOVA using origin as a factor. *N*_e_ was taken from the formula 1/(1-*H*_E_) and then tested with the non-parametric Tukey test [33]. *N*_e_ was used instead of (*N*_a_), considering that it is less sensitive to rare alleles and sample size. *H*_E_ was taken from the formula *H*_E_ = 1- (Σ*q*_i_^2^), where *q*_i_ represents the frequency of the *i* ^th^ allele in the population. *H*_E_ was converted into 1/*H*_E_ and then tested with the non-parametric Kruskal Wallis test. All statistical analyses were performed using R software v.3.3.2. The significance level was set below 0.001 (*P* < 0.001) to minimise sources of uncertainty.

To evaluate the genetic structure of populations, we relied on multiple approaches: Principal Coordinate Analysis (PCoA), Neighbour Joining Tree, AMOVA analysis, measurement of the index of differentiation (*F*_ST_) and use of a non-spatial Bayesian algorithm. These approaches were chosen in order to obtain a broad view of the genetic structure of this invasive species in Italy. PCoA, obtained with GenAIEx software, was used to display genetic divergence across *D. suzukii* in a multidimensional space, considering frequency data. Unrooted Neighbour Joining Tree based on Nei’s genetic distance constructed using DARwin software was complementary to PCoA analysis [34]. AMOVA analysis obtained using the Arlequin v.3.5 [35] program was performed to estimate variability distribution within and between the tested groups. The level of genetic differentiation in populations was detected using the *F*_ST_ values obtained with Microsatellite Analyzer (MSA) v.4.05 software [19]. The program allows comparison of each observed *F*_ST_ value with that obtained in 10,000 matrix permutations in order to define the statistical significance of each *F*_ST_.

The Bayesian method was implemented with STRUCTURE software v.2.3.3 [36, 37]. This program was employed in order to obtain clusters of individual genotypes. The analysis was run using the admixture hypothesis, which is based on correlated allele frequencies, in which each sample contains a portion of the genome of each ancestral population. This, correlated to the allele frequency model, allows calculation of the log likelihood for the data, L(K). Not knowing the origin and the degree of isolation of the studied populations a priori, this model is considered to be the most appropriate in these situations [36]. Prior probability, i.e. the probability that an individual belongs to any K reference populations, is defined as l/K. The K value was fixed from 1 to 10 with 20 replicates of each K to test the convergence of the Markov chain. A total of 1000,000 simulations per run and 500,000 Markov Chain Monte Carlo MCMC repetitions were fixed. Once the results were obtained, they were scored with STRUCTURE HARVESTER software to detect the number of K groups that best fit the dataset according to the Evanno test [38, 39]. GENECLASS v.2.0 [40] was run to estimate the probability of each individual in a population belonging only to that population, the probability of it being an immigrant from each of the other populations, and the probability of it being a migrant to the other populations. BOTTLENECK v.1.2.02 [41] was run in order to evaluate whether demographic events such as population contraction or expansion took place in each population.

Heterozygosity excess, which is associated with a population expansion, was tested with the two-phase mutation model (TPM) using Wilcoxon signed-rank test, which according to Piry et al. is the most appropriate and powerful test when dealing with less than twenty loci [41]. Parameters were set as 20% multiple-step mutations and 80% single-step mutations with 1000 iterations. In order to verify the effect of isolation by distance, and therefore to find possible correlation between genetic and geographical distances, the ISOLDE option in GENEPOP software was run.

## Results

### Genetic diversity

The variability indices of the 15 SSR loci are shown in Table 1. The number of alleles per locus across populations ranged from 8 (DS17) to 20 (DS07), with an average (± standard deviation) of 13.6 ± 3.37. The PIC estimate ranged from 0.68 (DS14) to 0.84 (DS07), suggesting that this set of loci is informative for population analysis. Only five alleles were in Hardy-Weinberg Equilibrium (DS07, DS09, DS22, DS23, DS26), while the other 10 showed significant HWE deviations, with nine loci having an excess of *H*_O_ (Table 1). The reason for the HWE disequilibrium could be the presence of null allele that may affect estimation of population differentiation [42, 43]. The mean *H*_O_ across loci ranged from 0.68 (DS32) to 0.91 (DS16), while *H*_E_ ranged from 0.71 (DS14) to 0.86 (DS07). Mean *H*_O_ across populations ranged from 0.66 ± 0.16 (Trentino2), to 0.89 ± 0.09 (Tuscany) (Table 2). Allelic richness ranged from 6.23 in Trentino1 to 8.58 in Apulia. For most of the loci the *F*_IS_ was negative. *F*_IS_ values ranged from −0.28 in Sicily to 0.07 in Trentino2. In the Additional file 1 are reported all the data concerning the observed and expected heterozygosity, the number of alleles, the effective number of alleles, the number of private alleles, the F-statistic (*F*is, *F*it and *F*st) and the fixation index. The Tukey test revealed a significant effect of population origin on the heterogeneity of *N*_e_ on comparing the following sampled populations: Trentino1 and Apulia, Trentino2 and Apulia, Sicily and Apulia, and Sicily and Tuscany (*P* < 0.001). On analysing all the populations together with ANOVA, using the collection site as a factor, *N*_e_ showed a significant difference between populations (*F* = 3.86, *P* < 10^−10^). The effect of the collection site was also evident in mean *H*_E_ (F 4.19, P < 0.001).

**Table 1:** Summary of the genetic variability at 15 microsatellite loci

Locus	Repeat	PIC	Allele range	*N*	*H*_O_	*H*_E_	H-W
DS05	(TG)10	0.74	250–284 bp	11	0.79	0.77	**
DS07	(CA)13	0.84	180–220 bp	20	0.89	0.86	NS
DS08	(AG)10	0.81	118–158 bp	17	0.84	0.83	***
DS09	(AC)15	0.72	200–230 bp	13	0.71	0.75	NS
DS14	(TG)10	0.68	136–239 bp	11	0.75	0.71	**
DS15	(GT)11	0.76	238–278 bp	13	0.88	0.79	*
DS16	(AC)13	0.78	85–119 bp	15	0.91	0.81	***
DS17	(GT)10	0.70	93–113 bp	8	0.90	0.74	***
DS20	(AG)12	0.74	207–235 bp	13	0.84	0.77	*
DS22	(GT)11	0.71	304–334 bp	13	0.83	0.75	NS
DS23	(AC)10	0.75	236–266 bp	13	0.76	0.77	NS
DS25	(CA)10	0.71	222–280 bp	18	0.74	0.74	***
DS26	(CA)10	0.73	79–109 bp	10	0.76	0.77	NS
DS28	(TG)11	0.78	141–161 bp	11	0.86	0.81	***
DS32	(TG)15	0.83	310–376 bp	18	0.68	0.85	***

Repeat, motive of the microsatellite; *PIC* polymorphic information content; Allele range, *N* number of alleles, *H*o,observed heterozygosity, *H*e expected heterozygosity

**Table 2:** Level of genetic diversity across 9 populations of *D. suzukii*

	*N*_a_	*N*_e_	*A*_r_	*N*_p_	*A*_p_	*H*_O_	*H*_E_	*A*_n_	*F*_IS_
Trentino1	6.40 ± 1.29	3.65 ± 0.93	6.23	1	0.06	0.68 ± 0.18	0.70 ± 0.07	0.01	0.02
Trentino2	7.60 ± 1.50	3.83 ± 1.01	7.36	8	0.53	0.66 ± 0.16	0.72 ± 0.08	0.03	0.07
Sardinia	7.87 ± 1.40	4.37 ± 0.80	7.69	6	0.40	0.82 ± 0.10	0.76 ± 0.05	0.03	−0.08
Latium	7.80 ± 1.37	4.18 ± 0.87	7.44	6	0.40	0.83 ± 0.13	0.74 ± 0.06	0.04	−0.11
Sicily	5.60 ± 0.98	3.25 ± 0.58	5.44	1	0.06	0.87 ± 0.11	0.68 ± 0.05	0.11	−0.28
Apulia	8.87 ± 3.44	5.22 ± 2.53	8.58	25	1.66	0.81 ± 0.13	0.77 ± 0.07	0.01	−0.04
Tuscany	7.47 ± 1.12	4.68 ± 0.98	7.23	2	0.13	0.89 ± 0.09	0.77 ± 0.04	0.06	−0.14
Liguria	7.40 ± 1.50	4.41 ± 0.77	7.11	3	0.20	0.87 ± 0.10	0.76 ± 0.03	0.05	−0.13
Veneto	7.53 ± 1.30	4.14 ± 0.50	7.26	5	0.33	0.83 ± 0.16	0.75 ± 0.02	0.04	−0.10

*N*_a_ mean number of alleles, *N*_e_ mean effective number of alleles, *A*_r_ mean of allele richness, *N*_p_ number of private alleles, *A*_p_ mean frequency of private alleles, *H*_O_ mean observed heterozygosity, *H*_E_ mean expected heterozygosity, *A*_n_ mean frequency of null alleles, *F*_IS_ mean inbreeding coefficient

### Genetic population structure and gene flow

**Fig. 3:**
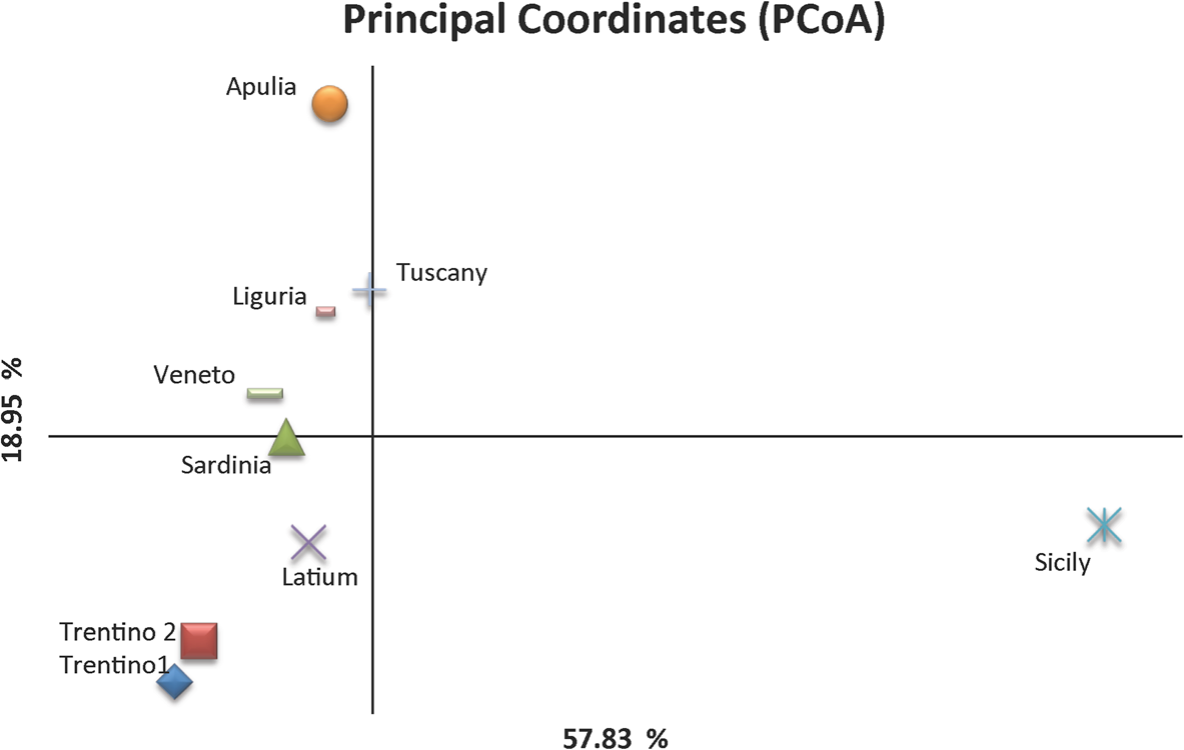
Principal coordinate analysis (PCoA) of nine populations generated from genetic distance calculation in GenAIEx program

**Fig. 4:**
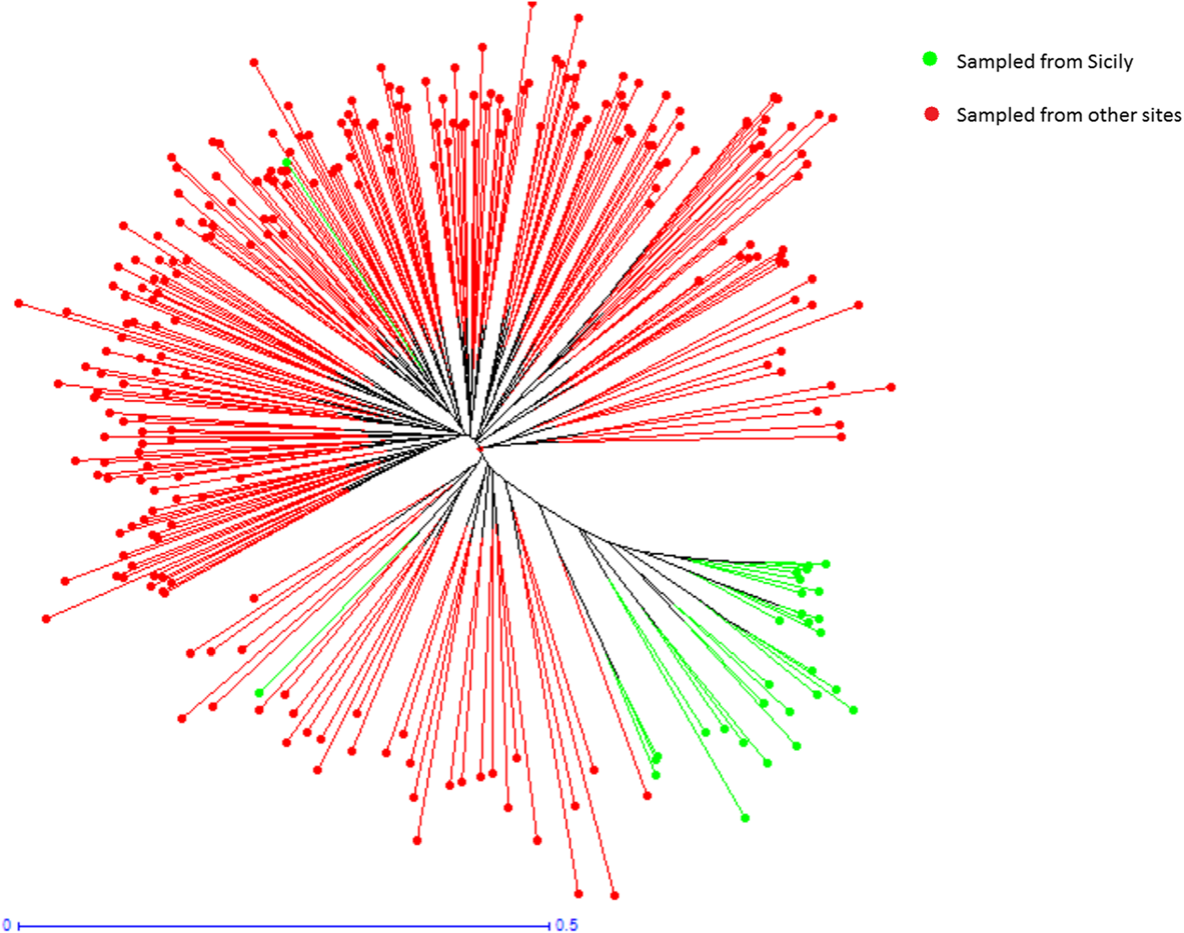
Unrooted neighbour joining (UNJ) tree obtained from DARwin software. Each brunch represents single individual

An estimate of variability distribution (AMOVA) within the tested populations indicated that 96% of the variation occurred within individuals, while only 4% of total variation was detected between populations. Table 3 gives a summary of analysis of variance for the nine *D. suzukii* populations. The results of PCoA are shown in Fig. 3. The first axis explains 57.9% of genetic variation, while the second axis explains 18.9%. The first axis separates the Sicilian population from the remaining populations. The second axis mainly divides Apulia, Tuscany, Liguria and Veneto from the others. The unweighted Neighbour-Joining dendrogram represented in Fig. 4 supports data obtained using PCoA: the Sicilian group has the same origin as the other populations, but individuals belong to a separated cluster.

**Table 3:** Analysis of molecular variance test (AMOVA)

Source of variation	DF	SS	VC	%PV
Among Populations	8	165.16	0.24	4%
Within Individuals	278	167.05	6.00	96%

*DF* degree of freedom, *SS* sum of squares, *VC* variance components, *%PV* percentage of total variation

**Fig. 5:**
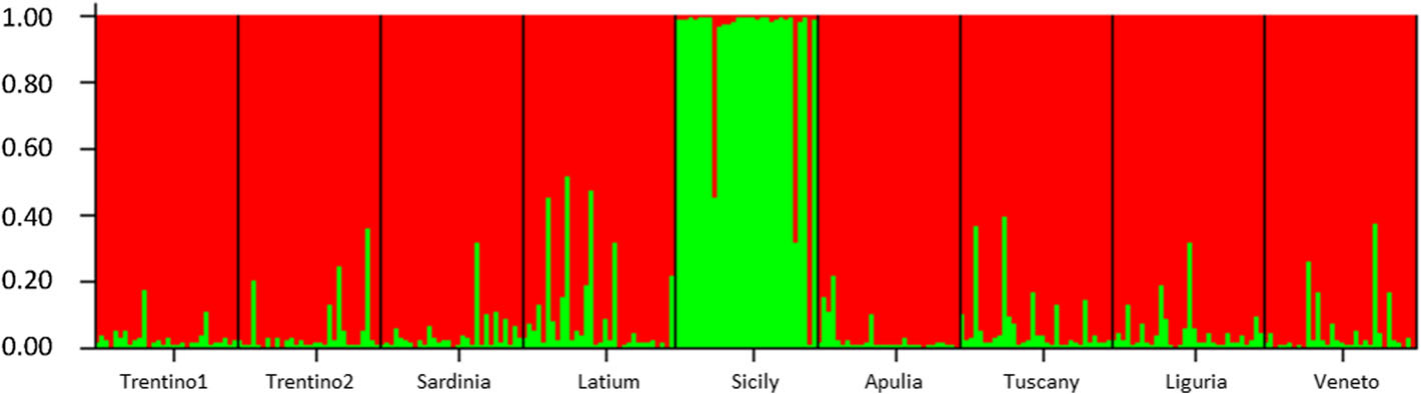
Genetic structure of nine Italian *D. suzukii* populations estimated by structure analysis

The *F*_ST_ values confirmed the genetic differentiation between the Sicilian group and the others. Considering all the populations, 30 of the 36 pairwise comparisons tested were significantly different from zero (Table 4). The least significant differentiation was between Liguria and Veneto (*F*_ST_ = 0.003), while the greatest divergence was between Sicily and Trentino1 (*F*_ST_ = 0.135). Population structure analysis led to the identification of two clusters (K = 2), based on the Evanno method (Fig. 5) and revealed genetic homogeneity between most populations, with the exception of flies collected in Sicily. The data of gene flow are reported in Table 5. The findings show that the Sicilian population did not migrate significantly to any other populations (m < 0.100). Although there was no gene flow from Sicily to the other regions, migrant flow occurred from Trentino2 (m = 0.100), Sardinia (m = 0.176), Lazio (m = 0.281), Tuscany (m = 0.195), and Liguria (m = 0.111). On the other hand, migration occurred from Apulia to other regions, but not to Apulia (m < 0.100) from other regions. Excluding Sicily and Apulia, the remaining seven populations both received and provided significant genetic information in relation to other populations (m > 100). Comparisons of Trentino2 and Veneto (m = 0.430 and m = 0.485 respectively), Sardinia and Tuscany (m = 0.316 and m = 0.327 respectively) and Lazio and Liguria (m = 0.376 and m = 0.315 respectively) revealed a similar migration rate in both directions. Considering the likelihood of the presence of migrants across populations, nine migrants (seven females and two males) were detected with a probability of less than 0.01.

**Table 4:** Pairwise *F*st among the nine populations of *D. suzukii*

	Trentino1	Trentino2	Sardinia	Latium	Sicily	Apulia	Tuscany	Liguria	Veneto
Trentino1	0.000								
Trentino2	0.006	0.000							
Sardinia	**0.022**	**0.011**	0.000						
Latium	0.009	0.007	**0.017**	0.000					
Sicily	**0.135**	**0.121**	**0.109**	**0.097**	0.000				
Apulia	**0.051**	**0.040**	**0.038**	**0.036**	**0.116**	0.000			
Tuscany	**0.039**	**0.031**	**0.015**	**0.022**	**0.097**	**0.038**	0.000		
Liguria	**0.026**	**0.017**	**0.010**	**0.018**	**0.096**	**0.033**	**0.015**	0.000	
Veneto	**0.022**	0.006	0.008	**0.012**	**0.113**	**0.036**	**0.017**	0.003	0.000

Boldface indicates values significantly different from zero (*P* = 0.05)

**Table 5:** Individual assignment analysis obtained using GENECLASS

	Trentino1	Trentino2	Sardinia	Latium	Sicily	Apulia	Tuscany	Liguria	Veneto
Trentino1	**0.391**	**0.512**	**0.518**	**0.604**	0.004	**0.272**	**0.344**	**0.482**	**0.485**
Trentino2	**0.288**	**0.477**	**0.492**	**0.524**	0.008	**0.307**	**0.394**	**0.506**	**0.485**
Sardinia	0.087	**0.254**	**0.444**	**0.270**	0.005	**0.138**	**0.327**	**0.305**	**0.301**
Latium	**0.149**	**0.386**	**0.349**	**0.472**	0.008	**0.194**	**0.324**	**0.315**	**0.270**
Sicily	0.015	**0.100**	**0.176**	**0.281**	**0.611**	0.064	**0.195**	**0.111**	0.089
Apulia	0.008	0.084	0.051	0.056	0.001	**0.377**	0.049	0.040	0.053
Tuscany	0.052	**0.162**	**0.316**	**0.209**	0.006	**0.115**	**0.567**	**0.266**	**0.176**
Liguria	**0.109**	**0.362**	**0.423**	**0.376**	0.004	**0.204**	**0.388**	**0.466**	**0.344**
Veneto	**0.183**	**0.430**	**0.454**	**0.456**	0.006	**0.283**	**0.401**	**0.473**	**0.564**

Boldface indicates significant migration rate values (m ≥ 100)

Results from the individual population analysis using the TPM model did not support an expansion scenario. On the contrary, population bottleneck, defined by significant heterozygote deficiency was present in the Trentino2 population (*P* = 0.004). The nine populations showed no significant correlation when comparing genetic and geographical distances [*R* ^2^ = 0.014, *P* = 0.568, *F*_ST_/(1-*F*_ST_) = 0.049 + Ln (geographical distance) = 0.001].

## Discussion

### Genetic diversity

The introduction of invasive species to new environments poses threats to biodiversity, agriculture, public health and ecosystem integrity [44–47]. For this reason, considerable attention is paid to the rapid spread of alien species [46, 48]. Genetic characteristics deeply affect the capacity for expansion [49]. Therefore, in order to mitigate their impact and define management strategies it is imperative to study these fundamental characteristics. Currently techniques such as genomics [50–52], transcriptomics [53, 54], and metagenomics [55, 56] allow us to investigate these basic traits.

This research investigated the genetic structure of *D. suzukii* collected in different areas of Italy. In particular, the aim of the analyses was to understand the gene flow of this species in a newly colonised environment. Our findings help to better understand the dynamics and complexity of this invasive species in Italy. The nine populations studied show a high level of genetic variation. The high number of alleles per locus detected clearly demonstrated the discriminatory power of these markers. Taking into consideration *N*_e_, *H*_E_ and *H*_O_, it is evident that the level of genetic differentiation is similar in *D. suzukii* collected across Italy, even in the locations at the greatest distance from the likely spreading centre of the species in Italy [25]. The high level of heterozygosity could be explained by good adaptation to new ranges due to a favourable environment, their reproductive power, and the absence or limited presence of natural competitors and predators [57, 58]. Bahder et al. found that populations from Washington were much less polymorphic than those in California, suggesting a recent strong population bottleneck associated to the recent invasion of the former [22]. Washington has a much cooler climate than California, similar to the contrast between Trentino and the rest of Italy. However, we did not observe such a contrast in heterozygosity, probably due to the highly favourable habitat found in Trentino coupled with a high migration rate with the rest of the Italian populations. Heterozygosity deficiency was detected for one out of the nine investigated populations. In contrast, the remaining eight groups showed heterozygosity excess. Sicily in particular had the lowest heterozygosity value (−0.28). Negative results indicate random mating, therefore a lack of inbreeding among the collected individuals. In contrast, Trentino1 and Trentino2 had a positive *F*_IS_ value, indicating inbreeding. The Apulian population showed the greatest number of private alleles (25). This could be the consequence of a steady introduction of new alleles due to migration, possibly associated with human-mediated transport [25, 59].

### Genetic structure analysis

Moderate genetic differentiation between most of the groups was in evidence for the nine populations, while the Sicilian population was the most differentiated from the others. This is supported by the NJ tree, PCoA data and structural analysis. At the same time, low differentiation between the other populations may be due to gene flow, which can homogenize gene frequency across populations. Data concerning the reduction or expansion of the studied populations indicated that Trentino2 was the only group having indices of genetic bottleneck.

### Migration pattern

Human transportation is the most probable explanation for the extensive spread of *D. suzukii*. [25, 59]. When an alien species is introduced into an environment outside its native range, expansion can be identified not only by analysing genetic diversity indices, but also by analysing the genetic flow between populations, which is a direct proof of rapid distribution. [60, 61]. In particular, in the last 40 years, the risk of biotic invaders has increased significantly because of levels of international trade not seen before [62]. This situation facilitates genetic flow between groups located in different locations, and may well apply to the results of our study. For instance, the observation that the level of heterozygosity (*F*_st_) does not clearly decline (increase) from the hypothetical source population (Livorno, Tuscany) and that there is a high migration rate among localities, suggests that *D. suzukii* moves extensively across most of the Italian peninsula. Most of the Sicilian production of vegetables and fruit, including high *D. suzukii* susceptible hosts, is frequently exported to central and northern Italy. While this could suggest a high probability of flies being transported between Sicily and the rest of the peninsula, our results indicate that there was no gene flow from Sicily to other regions. This is probably due to the fact that ripe fruits are exported from Sicily mostly during the cold season, when moderate temperatures allow the production of berry fruit in Sicily, but not in the rest of Italy. Therefore, any *D. suzukii* accidentally moving from Sicily to the rest of Italy would arrive at a time when the local population is made up of a few individuals in winter diapause [27].

A second interesting piece of information revealed by our results is related to the scenario in Sardinia. To satisfy local demand for berry fruit, this region imports fruit from Italy and northern Europe, Spain, the USA and South America. The flies used in this study were collected in Arborea, a town 13 km away from the port of Oristano, one of the most important commercial ports in Italy. Thus, it is likely that the Sardinian population is made up of immigrants from other regions, as suggested by the low differentiation between this population and those on the mainland.

## Conclusion

This research represents the first study investigating the pattern of genetic variability for *D. suzukii* following its introduction to Italy. Defining the population structure of a species, in particular of an invasive species, it is necessary not only to improve our knowledge of the genetic architecture, but also to apply knowledge. Indeed, understanding the current genetic structure of *D. suzukii* has significant implications in relation to geographical and economic impact. The evaluation of the genetic status of the *D. suzukii* populations in newly invaded areas and their expansion or reduction phases during defined periods of the year, may thus provide valuable information for predicting population spread, outbreaks, and improve integrated pest management programmes. Proper genetic management practices for *D. suzukii* and constant monitoring are therefore critical for maintaining populations under control.

The information obtained can be applied in particular to the management of coastal areas; one important action could be to increase monitoring control with the use of traps and other early warning tools in order to limit either multiple reintroductions of the same species or new introductions of exotic organisms.

## Additional file

Additional file 1: Data explained allele frequencies for each locus at each location. Are in more reported the observed and expected heterozygosity, the number of alleles, the effective number of alleles, the number of private alleles, the F-statistic (*F*is, *F*it and *F*st) and the fixation index. (XLSX 246 kb)
